# Superior Mesenteric Artery Thrombosis as a Complication of Polycythemia Vera: A Case Report

**DOI:** 10.3390/reports9020109

**Published:** 2026-04-01

**Authors:** Ljiljana Milić, Dragana Arbutina, Radosav Radulović, Marko Šurlan, Aleksandar Karamarkovic

**Affiliations:** 1 Clinic for Surgery “Nikola Spasic”, University Clinical Hospital Center “Zvezdara”, 11 000 Belgrade, Serbia; ljilja.milic@gmail.com (L.M.); raderadulovic89@gmail.com (R.R.); markosurlan1995@gmail.com (M.Š.); alekara@sbb.rs (A.K.); 2 Faculty of Medicine, University of Belgrade, 11 000 Belgrade, Serbia

**Keywords:** polycythemia vera, superior mesenteric artery thrombosis, acute mesenteric ischemia, myeloproliferative neoplasms

## Abstract

**Background and Clinical Significance:** Polycythemia vera (PV) is a myeloproliferative neoplasm associated with a markedly increased risk of arterial and venous thrombosis. Superior mesenteric artery (SMA) thrombosis is an exceptionally rare but potentially fatal complication. **Case Presentation:** We report the case of a 25-year-old man with previously diagnosed, JAK2-negative PV who presented with acute abdominal pain, nausea, vomiting, abdominal distension, and absence of stool and flatus, consistent with clinical features of intestinal obstruction. Laboratory testing revealed marked leukocytosis, elevated inflammatory markers, and subtherapeutic anticoagulation (INR 1.2) despite ongoing oral therapy. Multislice computed tomography demonstrated occlusion of the SMA with developed collateral circulation and features of small-bowel ischemia. Due to progression to an acute abdomen, emergency laparotomy was performed, revealing jejunal perforation with preserved viability of the remaining bowel. Primary closure was carried out, followed by peritoneal lavage and drainage. The postoperative course was uneventful. After correction of anticoagulation and therapeutic INR monitoring, no recurrent thrombotic events were observed during follow-up. **Conclusions:** This case underscores the importance of strict anticoagulation control, early imaging, and prompt surgical intervention in patients with PV, even in young individuals and in atypical vascular territories.

## 1. Introduction and Clinical Significance

Polycythemia vera (PV) is a myeloproliferative neoplasm first conceptualized in 1951 by William Dameshek [1]. It is characterized by increased production of blood cells, particularly erythrocytes, an increased tendency toward thrombosis and hemorrhage, heterogeneous clinical manifestations, and a cumulative risk of progression to myelofibrosis and/or transformation into acute myeloid leukemia [2]. The estimated incidence is 2.3–2.8 per 100,000 individuals per year, with a median age at diagnosis of approximately 60 years and a male-to-female ratio of 1.2:1 [2].

Thromboembolic events represent the main complication of myeloproliferative neoplasms and the leading cause of morbidity and mortality in patients with PV [3,4,5]. Thrombosis is reported to occur in approximately 39–41% of patients during the disease course [6,7]. The JAK2V617F mutation plays a crucial role in the development of thrombotic complications. Thrombogenesis in PV is multifactorial and involves abnormalities of platelets, erythrocytes, and leukocytes, as well as endothelial dysfunction [7,8]. Additional contributors include elevated hematocrit and blood viscosity, increased expression of adhesion molecules, and enhanced inflammatory activity associated with the JAK2 mutation [9,10].

Thrombotic complications in polycythemia vera most commonly involve the coronary, cerebral, and peripheral arterial circulation, while venous events frequently occur as deep vein thrombosis, pulmonary embolism, or splanchnic venous thrombosis. In contrast, superior mesenteric artery (SMA) thrombosis represents an extremely rare but life-threatening complication of the disease. Superior mesenteric artery (SMA) thrombosis represents an extremely rare but life-threatening complication of polycythemia vera. Acute mesenteric ischemia caused by arterial thrombosis is associated with very high mortality rates, particularly when diagnosis and treatment are delayed [11,12]. Due to nonspecific clinical presentation and low incidence, the diagnosis is frequently established late, which further worsens outcomes. Reports of such rare cases are clinically important, as they emphasize the need for a high index of suspicion for thrombotic complications in patients with myeloproliferative neoplasms, even in atypical locations. The aim of this report is to present a case of superior mesenteric artery thrombosis as a complication of polycythemia vera, with emphasis on diagnostic and therapeutic challenges and a review of relevant literature.

## 2. Case Presentation

A 25-year-old male patient was admitted to the Department of Hematology due to severe diffuse abdominal pain accompanied by nausea, vomiting, absence of stool, and elevated body temperature that had developed one day prior to admission.

The patient was initially examined by a surgeon and a urologist because of nonspecific abdominal pain. At that time, there were no indications for emergency surgical treatment. Analgesic and antibiotic therapy was administered, and outpatient follow-up was recommended.

Polycythemia vera had been previously diagnosed in 2017 based on bone marrow biopsy and clinical criteria, according to the available medical documentation. According to available medical information, JAK2 V617F mutation testing was negative; documentation regarding exon 12 testing was not available. The patient had undergone repeated therapeutic phlebotomies and, at the time of admission, was receiving oral anticoagulant therapy with acenocoumarol. He was an active smoker, had no comorbidities and no positive family history of thrombosis, and reported an aspirin allergy.

On admission, blood pressure was 120/80 mmHg. The patient was conscious, oriented, and ambulatory, with elevated body temperature at symptom onset. The skin and visible mucous membranes were hyperpigmented. He was asthenic, with no hemorrhagic syndrome or lymphadenopathy. Abdominal examination revealed a distended abdomen with infraumbilical tenderness and reduced peristalsis. Detailed documentation of heart rate, oxygen saturation, and serum lactate levels was not available in the accessible medical records, which represents a limitation of this report.

Laboratory analyses demonstrated erythrocytosis (7.26 × 10^12^/L), leukocytosis (26.6 × 10^9^/L) with neutrophilia, markedly elevated C-reactive protein (241.6 mg/L), elevated D-dimer (4.27 mg/L FEU), mildly prolonged prothrombin time, and positive fibrin degradation products. INR at presentation was 1.2, indicating subtherapeutic anticoagulation. Detailed laboratory findings at admission are presented in Table 1.

Plain abdominal radiography showed no pneumoperitoneum but marked dilatation of intestinal loops.

Abdominal ultrasonography revealed splenomegaly, numerous collateral veins in the mesentery and perisplenic region, dilated small bowel loops, and free fluid in the pelvis.

Multislice computed tomography of the abdomen and pelvis showed mild hepatomegaly with heterogeneous parenchyma and no focal lesions. The spleen was enlarged (15.6 × 6.3 cm) and lacked normal post-contrast enhancement in both arterial and venous phases. The abdominal aorta was of normal morphology. The celiac trunk was gracile, with thrombosis of the splenic artery approximately 17 mm from its origin. The left hepatic artery originated directly from the celiac trunk and was suboccluded over a 25 mm segment. The right hepatic artery originated from the superior mesenteric artery and showed no significant abnormalities. The superior mesenteric artery was occluded approximately 35 mm from its origin. Developed collateral branches supplying the intestinal loops were observed. The splenic vein could not be clearly visualized. Dilated small bowel loops with air–fluid levels, segmental bowel wall edema up to 8 mm, and multiple mesenteric, paraaortic, paracaval, and parailiac lymph nodes were noted. Venous collateral vessels were visible in the pelvis and near the inferior pole of the spleen.

Initially, the patient was treated with broad-spectrum antibiotics (ceftriaxone and ciprofloxacin), volume resuscitation, low-molecular-weight heparin, and therapeutic phlebotomy with evacuation of approximately 300 mL of blood. Phlebotomy was performed due to underlying polycythemia vera and elevated erythrocyte count, aiming to reduce blood viscosity and potential thrombotic progression.

During hospitalization, due to progression to an acute abdomen, the patient was transferred to the Department of Surgery and underwent emergency laparotomy after short preoperative preparation.

Midline laparotomy revealed a small amount of turbid fluid with fibrin deposits on the small bowel. Jejunal perforation was identified approximately 30 cm distal to the ligament of Treitz. The remaining small and large bowel were viable. Numerous dilated and engorged collateral vessels of the central colic circulation were observed. Primary closure of the jejunal perforation was performed using interrupted sutures, followed by extensive peritoneal lavage and drainage.

The postoperative course was uneventful. Intestinal passage was restored, oral feeding was initiated, and the patient was discharged on postoperative day 14 in good general condition. Chest CT was unremarkable.

After discharge, treatment was continued in cooperation with the attending hematologist. Oral anticoagulant therapy was resumed with concomitant low-molecular-weight heparin until a therapeutic INR was achieved (INR 3.8).

Thrombophilia testing revealed no mutations of factor II, factor V Leiden, or PAI-1. Weakly positive lupus anticoagulant results were observed; however, testing was performed while the patient was receiving oral anticoagulation, which may have influenced the findings. The patient was heterozygous for an MTHFR variant, which is not considered an independent thrombotic risk factor.

At the most recent follow-up, conducted two days prior to manuscript revision, the patient remained clinically stable. Under regular hematological supervision and adjusted anticoagulation therapy with therapeutic INR monitoring, no further thrombotic or ischemic events were recorded. The chronological course of events is summarized in Table 2.

## 3. Discussion

### Study Limitations

Several limitations should be acknowledged. First, although the patient had previously been diagnosed with polycythemia vera based on bone marrow biopsy and clinical criteria, complete molecular documentation was not available. The JAK2 V617F mutation was reported as negative, while testing for JAK2 exon 12 mutations was not available in the accessible records. Therefore, the diagnosis of polycythemia vera cannot be considered fully secured according to current diagnostic criteria.

Second, some clinical parameters at presentation, including detailed hemodynamic data and serum lactate levels, were not available in the accessible medical documentation.

Third, the rarity of arterial mesenteric thrombosis in polycythemia vera and the limited number of published cases restrict broader conclusions regarding the exact pathophysiological mechanisms.

Thrombogenesis in patients with polycythemia vera (PV) is a complex and multifactorial process involving erythrocytosis, increased blood viscosity, inflammatory activation, endothelial dysfunction, and interactions among platelets, leukocytes, and the vascular endothelium. Although the JAK2V617F mutation is present in the majority of patients and has been associated with increased thrombotic risk, thrombotic events may also occur in JAK2-negative cases, indicating that the prothrombotic state in PV is not solely mutation-dependent [7,8,9,13].

Thromboembolic events and cardiovascular disease occur more frequently in patients with PV than in other myeloproliferative neoplasms and represent the main causes of morbidity and mortality [4,5,10]. A large population-based Swedish registry study demonstrated that, within three months after diagnosis, patients with PV had an approximately threefold increased risk of arterial thrombosis and a thirteenfold increased risk of venous thrombosis compared with matched controls [3,14].

Recent observational studies have shown that acute coronary syndromes and cerebrovascular events are among the most common arterial complications, whereas deep vein thrombosis, splanchnic venous thrombosis, pulmonary embolism, and superficial vein thrombosis are the most frequent venous manifestations [11,12,15,16]. In contrast, superior mesenteric artery (SMA) thrombosis remains an exceptionally rare arterial presentation.

The initial differential diagnosis in this patient included acute appendicitis, perforated peptic ulcer, mechanical small bowel obstruction, acute pancreatitis, and mesenteric venous thrombosis. However, severe abdominal pain disproportionate to physical findings, markedly elevated inflammatory markers, elevated D-dimer levels, and the patient’s known prothrombotic condition prompted early CT angiography, which confirmed arterial occlusion of the SMA.

In our patient, the primary manifestation of PV-related complications at the age of 25 years was extensive thrombosis of the splanchnic arterial circulation. Although the patient was receiving oral anticoagulation, the INR at admission was 1.2, indicating subtherapeutic anticoagulation. Inadequate anticoagulation control likely contributed to the development of arterial thrombosis. Active smoking may have represented an additional prothrombotic factor.

Mortality rates of acute thromboembolic occlusion of the superior mesenteric artery remain high, ranging from 60% to 90% [17,18,19,20,21]. Survival in this case may be attributed to early imaging, prompt surgical intervention, and the presence of well-developed collateral circulation observed on CT imaging.

We hypothesize that anatomical vascular variation, although not definitively confirmed, together with collateral development, may have contributed to limiting the extent of bowel ischemia. This interpretation remains speculative.

The role of therapeutic phlebotomy in the acute setting of arterial thrombosis is debated. In PV, maintaining hematocrit below 45% is recommended to reduce thrombotic risk. In this case, phlebotomy was performed to reduce blood viscosity and potential thrombotic propagation; however, such decisions require individualized clinical judgment and careful hemodynamic assessment.

Weakly positive lupus anticoagulant results were observed; however, testing was performed while the patient was receiving oral anticoagulation, which may lead to false-positive findings. Therefore, antiphospholipid syndrome could not be confirmed. Isolated MTHFR heterozygosity is not considered an independent risk factor for thrombosis.

Preventive strategies in PV include strict hematocrit control, consistent anticoagulation monitoring when indicated, smoking cessation, and individualized risk stratification [14]. At the most recent follow-up, no recurrent thrombotic events were observed after correction of anticoagulation and achievement of therapeutic INR levels, further underscoring the importance of strict anticoagulation control.

## 4. Conclusions

Superior mesenteric artery thrombosis represents an exceptionally rare but potentially fatal complication of polycythemia vera. This case underscores the importance of maintaining a high index of suspicion for acute mesenteric ischemia in patients with myeloproliferative neoplasms, even in young individuals and in the absence of typical vascular risk factors. Subtherapeutic anticoagulation may significantly increase the risk of catastrophic arterial events, highlighting the necessity of strict INR monitoring and individualized risk control. Early imaging, prompt surgical intervention, and multidisciplinary management are essential to improve outcomes. Well-developed collateral circulation may play a critical role in limiting ischemic injury and enabling survival.

Future studies involving larger cohorts of patients with myeloproliferative neoplasms are needed to better characterize rare arterial thrombotic complications and to clarify the mechanisms leading to unusual vascular involvement, such as superior mesenteric artery thrombosis. Improved molecular characterization of JAK2-negative cases may also help refine diagnostic certainty and thrombotic risk stratification.

## Figures and Tables

**Table 1:** Laboratory findings on admission.

Parameter	Result	Reference Range
Hemoglobin	13.4 g/dL	13.0–17.0
Hematocrit	49.6%	40.0–52.0
Erythrocytes	7.26 × 10^12^/L	4.0–5.5
Leukocytes	26.6 × 10^9^/L	4.8–10.8
Neutrophils (%)	82.0%	45.0–72.0
Neutrophils (absolute)	21.78 × 10^9^/L	1.9–8.0
Platelets	320 × 10^9^/L	150–400
C-reactive protein	241.6 mg/L	<5.0
D-dimer	4.27 mg/L FEU	<0.5
Fibrinogen	4.9 g/L	2.2–4.9
Prothrombin time	14.8 s	10.6–13.4
INR	1.2	0.8–1.1
aPTT	45.0 s	27.0–42.0
Creatinine	53 µmol/L	53–132
Urea	3.5 mmol/L	2.5–8.3
Albumin	32 g/L	35–52
Total protein	59 g/L	64–83

**Table 2:** Clinical timeline.

Time Point	Clinical Event
2017	Diagnosis of polycythemia vera based on bone marrow biopsy; JAK2 V617F reported negative.
2017–2019	Periodic therapeutic phlebotomies; oral anticoagulation with acenocoumarol.
Day −1	Onset of diffuse abdominal pain, nausea, vomiting, and absence of stool.
Day 0	Admission; laboratory findings significant for leukocytosis, elevated CRP and D-dimer; INR 1.2 (subtherapeutic).
Day 0	CT angiography demonstrated superior mesenteric artery occlusion with collateral circulation.
Day 0	Clinical deterioration; emergency laparotomy performed; jejunal perforation identified and repaired.
Postoperative course	Uneventful recovery; restoration of bowel function.
Postoperative day 14	Discharged in stable condition.
Follow-up (latest, 2 days prior to revision)	Clinically stable; no recurrent thrombotic events under therapeutic anticoagulation.

## Data Availability

The original data presented in the study are included in the article, further inquiries can be directed to the corresponding author.
